# Insights Into Cerebral Tissue-Specific Response to Respiratory Challenges at 7T: Evidence for Combined Blood Flow and CO_2_-Mediated Effects

**DOI:** 10.3389/fphys.2021.601369

**Published:** 2021-01-28

**Authors:** Allen A. Champagne, Alex A. Bhogal

**Affiliations:** ^1^Centre for Neuroscience Studies, Queen’s University, Kingston, ON, Canada; ^2^School of Medicine, Queen’s University, Kingston, ON, Canada; ^3^Department of Radiology, University Medical Center Utrecht, Utrecht, Netherlands

**Keywords:** cerebrovascular reactivity, 7T, temporal delays, CO_2_ sensitivity, RIPTiDe

## Abstract

Cerebrovascular reactivity (CVR) mapping is finding increasing clinical applications as a non-invasive probe for vascular health. Further analysis extracting temporal delay information from the CVR response provide additional insight that reflect arterial transit time, blood redistribution, and vascular response speed. Untangling these factors can help better understand the (patho)physiology and improve diagnosis/prognosis associated with vascular impairments. Here, we use hypercapnic (HC) and hyperoxic (HO) challenges to gather insight about factors driving temporal delays between gray-matter (GM) and white-matter (WM). Blood Oxygen Level Dependent (BOLD) datasets were acquired at 7T in nine healthy subjects throughout BLOCK- and RAMP-HC paradigms. In a subset of seven participants, a combined HC+HO block, referred as the “BOOST” protocol, was also acquired. Tissue-based differences in Rapid Interpolation at Progressive Time Delays (RIPTiDe) were compared across stimulus to explore dynamic (BLOCK-HC) versus progressive (RAMP-HC) changes in CO_2_, as well as the effect of bolus arrival time on CVR delays (BLOCK-HC versus BOOST). While GM delays were similar between the BLOCK- (21.80 ± 10.17 s) and RAMP-HC (24.29 ± 14.64 s), longer WM lag times were observed during the RAMP-HC (42.66 ± 17.79 s), compared to the BLOCK-HC (34.15 ± 10.72 s), suggesting that the progressive stimulus may predispose WM vasculature to longer delays due to the smaller arterial content of CO_2_ delivered to WM tissues, which in turn, decreases intravascular CO_2_ gradients modulating CO_2_ diffusion into WM tissues. This was supported by a maintained ∼10 s offset in GM (11.66 ± 9.54 s) versus WM (21.40 ± 11.17 s) BOOST-delays with respect to the BLOCK-HC, suggesting that the vasoactive effect of CO_2_ remains constant and that shortening of BOOST delays was be driven by blood arrival reflected through the non-vasodilatory HO contrast. These findings support that differences in temporal and magnitude aspects of CVR between vascular networks reflect a component of CO_2_ sensitivity, in addition to redistribution and steal blood flow effects. Moreover, these results emphasize that the addition of a BOOST paradigm may provide clinical insights into whether vascular diseases causing changes in CVR do so by way of severe blood flow redistribution effects, alterations in vascular properties associated with CO_2_ diffusion, or changes in blood arrival time.

## Introduction

Cerebrovascular tone is constantly being modulated to ensure adequate supply of oxygen and glucose to brain tissues (Paulson et al., 1990; Iadecola and Nedergaard, 2007). Control arterioles that branch from larger feeding arteries respond to local changes in metabolism, pH, or arterial CO_2_ partial pressure (P_a_CO_2_), work to regulate cerebral blood flow (CBF), ensuring proper delivery of nutrients and effective waste removal. This sensitive process, whereby dynamic changes in vascular tone modulate regional CBF in response to vasodilatory stimuli is defined as cerebrovascular reactivity [CVR; (Fisher et al., 2018; Liu et al., 2018)]. In recent years, CVR mapping has emerged as a robust clinical tool to understand the vascular physiology underlying conditions like stroke (Geranmayeh et al., 2015), moyamoya disease (Conklin et al., 2010), arterial stenosis (Mandell et al., 2008a), and traumatic brain injury (Len et al., 2011; Ellis et al., 2016; Champagne et al., 2020), as well as the hemodynamic processes associated with normal aging of cerebral tissues (De Vis et al., 2015; Bhogal et al., 2016).

Moving beyond amplitude-based assessments of vascular properties (i.e., CVR) advanced post-processing analyzes have emerged to characterize temporal markers associated with the CVR response (Duffin et al., 2015; Poublanc et al., 2015; Donahue et al., 2016; van Niftrik et al., 2017; Champagne et al., 2019). These markers can provide quantitative profiles that aid in the explanation of differences between vasodilatory response across cortical and sub-cortical tissues (Donahue et al., 2014; Thomas et al., 2014; Champagne et al., 2019) as well as pathological tissues with impaired or temporally altered hemodynamics due to collateralization (Donahue et al., 2016). The general consensus is that temporal characteristics of the vascular response reflect a combination of factors including arterial transit time, blood redistribution, and vascular response speed, which may be partially separated using a combination of hypercapnic (HC) respiratory challenges to stress the cerebral vasculature and hyperoxic (HO) respiratory challenges that have previously been implemented to act as endogenous contrast agents via O2-mediated changes in deoxyhemoglobin (Blockley et al., 2013; Champagne et al., 2019).

Through examinations of cerebrovascular architecture, it is known that cortical and deep gray-matter (GM) tissues have high arteriolar density (Moody et al., 1990), which are fed by larger vessels branching off major cerebral arteries (i.e., pial, lenticulostriate, and choroidal). During HC, arteriolar smooth-vessel mediated dilation decreases peripheral resistance in cerebral arteries leading to an increase in CBF through capillary beds in accordance with Poiseuille’s law (McDonald, 1974; Edvinsson et al., 2002). The bulk of these arterial resistance changes are in reaction to rising extravascular P_a_CO_2_ that, in turn, drives changes in blood pH and local vasodilatory mechanisms (Yoon et al., 2000; Tripp et al., 2001; Chesler et al., 2008). During MR imaging of HC, fractional changes in BOLD signal arise from the relationship between regional increases in blood flow and subsequent decreases in deoxy-hemoglobin concentration ([d-Hb]), which lengthens T_2_^∗^ relaxation (Ogawa et al., 1990, 1993). Assuming this coupling between CBF and [d-Hb] remains constant, differences in local CO_2_ accumulation, sensitivity and cross-membrane diffusion rate (Thomas et al., 2014), as well as the possible redistribution (or “vascular-steal”) effects between vascular networks (Conklin et al., 2010; Fierstra et al., 2010; Bhogal et al., 2015), may all bias CVR measurements derived using standard methods, emphasizing the need to include additional temporal marker for the cerebrovascular response.

While HC modulates the BOLD signal via changes in perfusion, the BOLD contrast can also be manipulated when combined with non-vasoactive HO breathing challenges (Blockley et al., 2013). As the arterial partial pressure of oxygen (P_a_O_2_) increases, complete hemoglobin saturation at the lungs drives the dissolution of abundant O_2_ into the blood plasma. This O_2_-loaded blood traverses the cardio-vascular network until it reaches the capillary beds where it diffuses toward cerebral tissues to meet metabolic demand. In terms of the BOLD contrast mechanisms, the increase in O_2_ supply results in a reduced unloading of O_2_ from arterial hemoglobin and a concomitant decrease in venous de-oxyhemoglobin content; this lengthens the blood T_2_^∗^ relaxation time, creating a similar BOLD signal response as what is observed during HC. This O_2_ based mechanism has been proposed as a means through which arrival time (Champagne et al., 2019) and cerebral blood volume (CBV) (Lu et al., 2003) can be assessed and assumes limited vasoconstriction and minor reduction in venous CBV (Bulte et al., 2007; Mark and Pike, 2012; Xu et al., 2012). Irrespective of potential vaso-constrictive effects, the bolus of highly saturated blood formed during HO breathing can act as a non-invasive endogenous contrast agent for estimation of bolus arrival time (Blockley et al., 2013; Liu et al., 2017; Champagne et al., 2019) since only minor reductions in blood velocity would be expected. Consequently, HO may be used to calibrate HC-derived CVR delays, and untangle physiological factors that contribute to the (patho)physiological mechanisms associated with diseases causing vascular impairments.

In MR-based methods that probe CVR using a gas delivery system (Slessarev et al., 2007; Mark et al., 2010; Fisher, 2016), HC is typically induced via inhalation of CO_2_ administered using a block or ramp respiratory challenge [see Figure 4 in Liu et al. (2018)]. Block-based HC is a stimulus where the end-tidal CO_2_ (P_ET_CO_2_) is rapidly increased from baseline in a boxcar function (typically +5–10 mmHg), which is assumed to evoke a dynamic CVR response (van der Zande et al., 2005). In contrast, the RAMP-HC stimulus involves a gradual increases in the P_ET_CO_2_ which is assumed to allow for more progressive changes in local P_a_CO_2_ (Ringelstein et al., 1988; Claassen et al., 2006; Battisti-Charbonney et al., 2011; Bhogal et al., 2014b). Although the step design is commonly used to characterize temporal CVR delays, no studies to date has yet to explore tissue-based differences in delays from the vascular response induced during a ramp design, and how those may differ from CVR delays derived from block designs. Based on the assumption that local CO_2_ levels are increased progressively during the ramp, it may be hypothesized that the slower arteriolar reactivity in response to decreasing pH allows for less blood flow redistribution and steal effects between vascular territories and tissues, that in turn, lead to shorter CVR delays. Additionally, given that HO-based BOLD mapping can be leveraged to estimate bolus arrival time, it may be hypothesized that a combined boxcar HC and HO respiratory stimulus can provide additional insight with respect to possible redistribution effects within local vessels, in the event that the region-specific lag times remain relatively increased compared to neighboring vascular territories.

In this study, we integrate HC- and HO-based respiratory challenges to gather insight about factors driving tissues-based differences in temporal delays of the CVR response. High spatial-resolution BOLD datasets were acquired at 7T using both BLOCK- and RAMP-HC paradigms to study the potential differences in CVR delays between dynamic and progressive local changes in CO_2_. Finally, a novel protocol using a combined HC and HO block, referred as the “BOOST” protocol, was used to study the effect of bolus arrival time on tissue-based CVR delays, in contrast to CO_2_ sensitivity.

## Materials and Methods

### Subjects

We retrospectively surveyed our seven Tesla subject database to identify subjects in which both RAMP- and BLOCK-HC designs were acquired using the same MR imaging readout. We were able to identify a total of nine subjects. In a subset of seven of these subjects, an additional paradigm was identified in which a HO–HC block (herein termed “BLOCK BOOST HC+HO”) was also administered. Age and gender information for the main (herein termed SAMPLE.1) and sub-group (herein termed SAMPLE.2) are provided in Table 1.

**TABLE 1:** Subject demographics and end-tidal measures.

**SAMPLE.1**	**(*N* = 9, 5 female)**	**Age: 30 ± 9 years**
Paradigm	Parameters	P_ET_CO_2_/O_2_ (mmHg)
BLOCK-HC	Baseline	36 ± 8 / 113 ± 1
	Delta	8 ± 1 / 6 ± 6
RAMP-HC	Baseline	35 ± 6 / 112 ± 11
	Min	31 ± 6 / 113 ± 10
	Max	51 ± 5 / 120 ± 16
	Delta	22 ± 6 / 9 ± 9

**SAMPLE.2**	**(*N* = 7, 3 female)**	**Age: 29 ± 6 years**

Paradigm	Parameters	P_ET_CO_2_/O_2_ (mmHg)
BLOCK-HC	Baseline	39 ± 6 / 111 ± 10
	Delta	8 ± 1 / 7 ± 6
RAMP-HC	Baseline	37 ± 5 / 111 ± 12
	Min	28 ± 6 / 112 ± 9
	Max	51 ± 4 / 121 ± 16
	Delta	20.8 ± 6 / 7 ± 7
BLOCK BOOST HC+HO	Baseline	39 ± 6 / 111 ± 10
	Delta	8 ± 1 / 159 ± 44

*Values are in mean ± standard deviation.*

### Respiratory Paradigms

**FIGURE 1:**
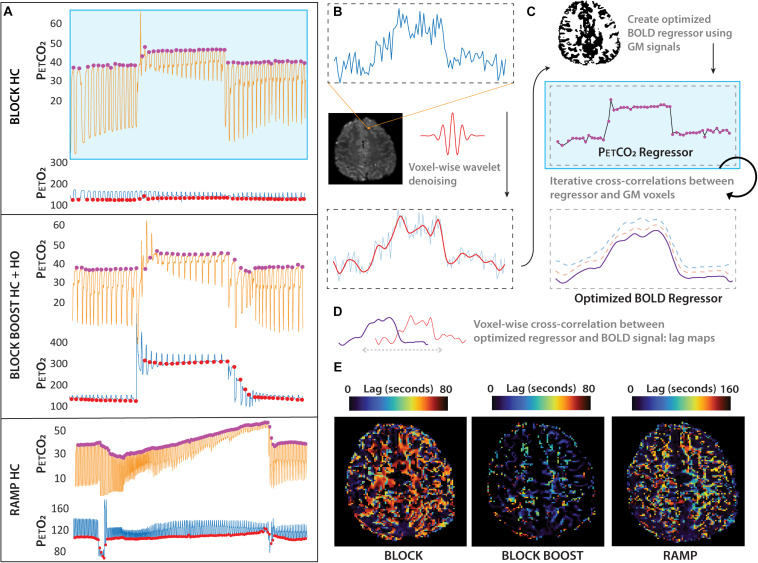
Summary schematic of the respiratory paradigms and imaging outputs. Sample data showing the RespirAct^TM^ (Toronto, ON, Canada) end-tidal trace **(A)** for CO_2_ (P_ET_CO_2_, mmHg; orange line and purple dot) and O_2_ (P_ET_O_2_, mmHg; blue line and red dot) during the hypercapnic (HC) and hyperoxic (HO) gas challenges used in this study: BLOCK HC only (top), BLOCK BOOST HC+HO (middle) and RAMP HC only (bottom). The Blood Oxygen Level Dependent (BOLD) signal acquired simultaneously **(B)** was denoised using a Bayesian wavelet approach on a voxel-by-voxel basis and then extracted from the gray matter **(C)** to compute the optimized BOLD regressor using the Rapid Interpolation at Progressive Time Delays (RIPTiDe) method. The final BOLD regressor was then cross correlated against the original denoised BOLD signal **(D)** at each voxel to compute voxelwise lag maps (seconds) **(E)**. This was repeated for each breathing challenges shown in **(A)**, for each subject.

Respiratory challenges were delivered using a 3rd generation RespirAct^TM^ (Thornhill Research Inc., Toronto, ON, Canada) system in combination with a rebreathing face mask. The mask was taped to ensure an air-tight seal using Tegaderm transparent dressing (3M, St. Paul, MN, United States). For both groups, the BLOCK-HC protocol consisted of a 90s increase in hypercapnia, preceded, and followed by a pre- and post-baseline period, respectively (Figure 1A). In the case of the combined BLOCK BOOST HC+HO experiment, a simultaneous increase in arterial O_2_ was implemented (Figure 1A). To ensure sharp transitions in arterial O_2_, the level of the O_2_ increase was limited such that the gas delivery system could evoke arterial changes within several breaths in line with the transitions possible when increasing arterial CO_2_ (Figure 1A). The progressive protocol (i.e., RAMP-HC) consisted of a baseline period followed by a 60 s hypocapnic period and a subsequent 5-min period of progressively increasing hypercapnia, ending with a return to baseline (Figure 1A). Ramped CO_2_ protocols are well suited to determine the precise vascular reserve capacity in a particular region. For example, impaired CVR will manifest as an early plateau in the BOLD-CVR response as vessels exhaust their dilatory reserve capacity. The progressive nature of the ramped stimulus means that the subject only experiences the high arterial CO_2_ levels required to elucidate mild impairments toward the end of the paradigm. In contrast, protocols based on BLOCK stimuli are suited to reveal dynamic aspects of the BOLD-CVR response and are imprecise in terms of revealing the dose-response profile of the CO_2_ stimulus. For this reason, CVR information can be obtained using a lower stimulus magnitude that spares the subject from prolonged high-level exposure that can potentially cause discomfort. With the exception of one subject, all three paradigms were acquired on the same day during the same scan session. The baseline and achieved end-tidal CO_2_ and O_2_ values across all paradigms used in our study are reported in Table 1. Typical end-tidal CO_2_ and O_2_ traces along with corresponding GM and WM ROI responses are shown for examples of each paradigm in Supplementary Figure 1.

### Magnetic Resonance Imaging

This study was approved by the medical research ethics committee of University Medical Center Utrecht (UMCU) and written informed consent was obtained from all subjects. The experiments were performed according to the guidelines and regulations of the WMO (Wet Medisch Wetenschappelijk Onderzoek). Subjects were scanned on a Philips 7 Tesla MRI system using a dual channel transmit coil in combination with a 32-channel receive coil. Image based (3rd order) shimming was performed. Respiratory challenges were administered throughout a multi-slice single-shot GE-EPI BOLD scan (flip angle: 90°, TR/TE 3000/25 ms, EPI/SENSE factor 47/3, reconstructed resolution: 1.5 mm × 1.5 mm, slice thickness: 1.6 mm, no slice gap, FOV: 217.6 mm × 192 mm, slices: 43, respiratory paradigm dependent scan duration: 507s for RAMP-HC and approx. 330s for BLOCK-HC/BOOST HC+HO).

### Computation of Vascular Reactivity and Temporal Lags

**FIGURE 2:**
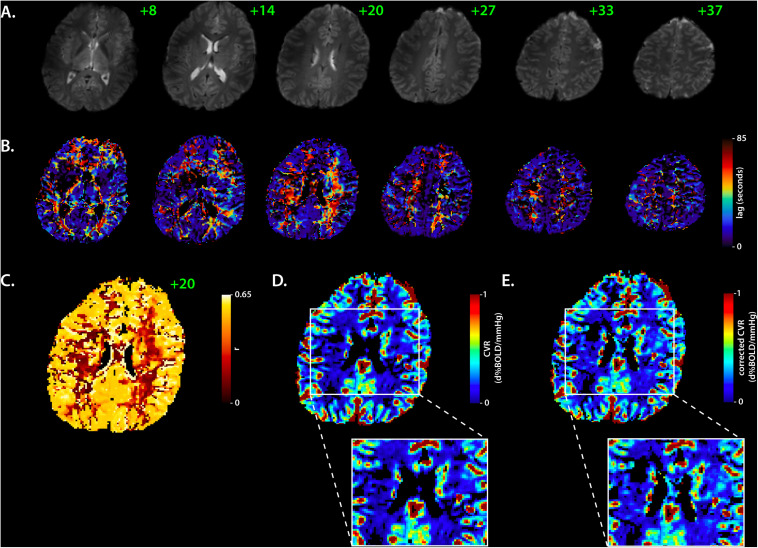
Corrected cerebrovascular reactivity mapping based on RIPTiDe lag times. Sample data showing BOLD EPI axial slices from the BLOCK-HC challenge in a single subject **(A)** used to compute the RIPTiDe lag maps **(B)**. The slice number is shown in green. In **(C)**, the matching voxelwise correlation coefficient (r) map (axial, 20) shows the fit between the BOLD signal and optimized RIPTiDe regressor. The original and lag-corrected cerebrovascular reactivity (CVR) maps are shown in **(D)** and **(E)**, respectively, with a specific look at deeper gray- and white-matter structures, to reflect the improvement in CVR following the lag time correction. Note that cerebrospinal fluid was removed from all maps to improve visual representation of the data. BOLD, Blood Oxygen Level Dependent, HC, hypercapnia, RIPTiDE, Rapid Interpolation at Progressive Time Delays.

Post-processing of the BOLD data consisted of temporal re-alignment [FSL:MCFLIRT; (Jenkinson et al., 2012)], segmentation [FSL:FAST; (Zhang et al., 2001)] and voxel-wise temporal de-noising using a Bayesian wavelet based approach (Figure 1B; symlet four wavelet, two levels, hard co-efficient threshold with level dependent noise estimation). End-tidal CO_2_ and O_2_ traces were resampled to the TR of the BOLD acquisition and aligned to the BOLD data based on the maximum correlation between the breathing trace and the individual mean GM signal. BOLD data and respiratory traces were then interpolated to a temporal resolution of 3000/8 ms (i.e., 8× oversampled) to account for temporal delays between slice acquisitions. The interpolated P_ET_CO_2_ traces were used as the initial probe to generate and optimized BOLD signal regressor for a correlation-based temporal lag analysis [RIPTiDe; (Frederick et al., 2012; Tong and Frederick, 2012); Figure 1C]. Based on voxel-wise lag estimates, individual lag maps were generated for the BLOCK-HC, RAMP-HC, and BLOCK BOOST HC+HO respiratory paradigms (Figure 1D). P_ET_CO_2_ traces as well as lag-adjusted traces were then used to generate CVR (Δ%BOLD/mmHg) and lag-adjusted CVR maps (seconds; Figure 2). This was accomplished by mapping the slope parameter obtained through linear regression of the lag adjusted P_ET_CO_2_ trace against the voxel-wise BOLD signal. Finally, BOLD data were spatially normalized to the 1-mm MNI152 atlas via *affine* and non-linear *bspline* transforms using elastix [version 5.0, (Klein et al., 2010; Shamonin et al., 2014)]. Transformation matrices were applied to CVR and lag-maps (using transformix) from each design to facilitate group region of interest analysis in segmented GM and WM tissues. Voxels containing less than four data points were thresholded from the MNI averaged CVR and lag maps.

### Comparisons of Vascular Reactivity and Temporal Delays Across Tissues and Paradigms

Segmented tissue-based probability maps were binarized from the 1-mm MNI152 atlas using a voxel-based threshold set to 50 and 90%, for the GM and WM, respectively. The tissue-specific masks were applied to the group-averaged CVR and lag maps to extract the parameter distribution for each respiratory design, along with mean and standard deviation (MATLAB, version 2019a, The Mathworks, MA, United States). This procedure was repeated for both SAMPLE.1 and SAMPLE.2, in order to assess the repeatability of the CVR and delay measurements from the BLOCK- and RAMP-HC. Tissue-based histograms for each average map were also assessed to look at the distribution of the CVR lags, between GM and WM.

## Results

### End-Tidal Measurements

All end-tidal measurements for each respiratory design are reported in Table 1. On average, participants were exposed to an 8 ± 1 mmHg increase in P_ET_CO_2_ during the BLOCK-HC, while maintaining changes in P_ET_O_2_ fluctuations well below physiological threshold. Similar increases in P_ET_CO_2_ were obtained during the BLOCK-BOOST (SAMPLE.2), with concurrent increases in P_ET_O_2_ averaged to 159 ± 44 mmHg, across subjects. During the ramp, larger increases in P_ET_CO_2_ were obtained, ranging between [20.8–22] ± 6 mmHg, with minimal changes in P_ET_O_2_.

### Cerebrovascular Reactivity and Temporal Delays

**FIGURE 3:**
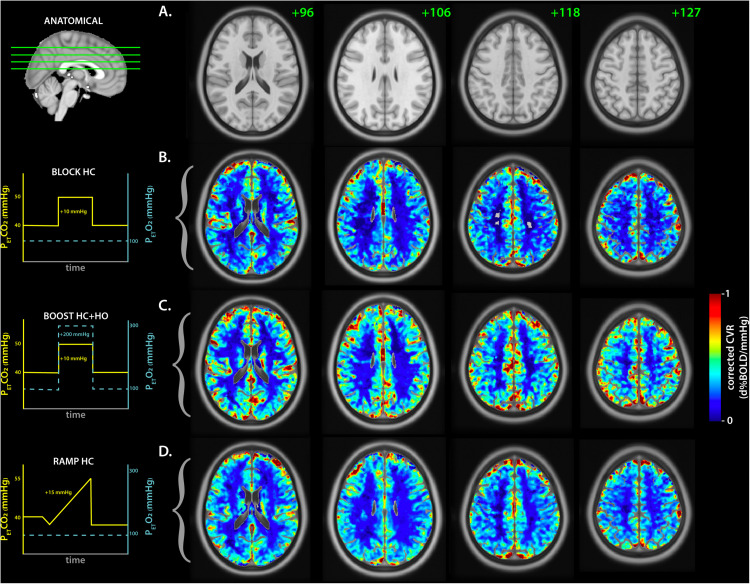
Average lag-corrected cerebrovascular reactivity maps for each respiratory challenge. **(A)** The anatomical axial reference slices for the MNI template are shown with the slice number in green. The matching sagittal frame showing the referenced frames is displayed on the left. The group-averaged lag-corrected cerebrovascular reactivity (CVR) maps are displayed for each respiratory challenge in **(B–D)**, representing the BLOCK-HC, BLOCK BOOST HC+HO, and RAMP-HC, respectively. A schematic of each respiratory design is shown on the left with the targeted end-tidal traces for CO_2_ (yellow) and O_2_ (cyan blue), as a reference.

All parameters described below are summarized in Table 2. Average CVR measurements for both GM and WM tissues were in agreement between the BLOCK- and RAMP-HC paradigms. In comparison, CVR measurements were higher during the BLOCK-BOOST, although relative differences between the GM and WM were maintained. Qualitatively, similar observations can also be made based on whole brain assessment of lag-corrected CVR (Figure 3).

**TABLE 2:** Gray- and white-matter average parameters for each stimulus.

**Stimulus**	**Parameters**	**GM**	**WM**
BLOCK HC (*N* = 9)	CVR (Δ% BOLD/mmHg)	0.38 ± 0.22	0.17 ± 0.11
	*r*^2^	0.49 ± 0.12	0.39 ± 0.09
	Lag (seconds)	21.80 ± 10.17	34.15 ± 10.72
RAMP HC (*N* = 9)	CVR (Δ%BOLD/mmHg)	0.38 ± 0.22	0.18 ± 0.10
	*r*^2^	0.54 ± 0.15	0.42 ± 0.12
	Lag (seconds)	24.29 ± 14.64	42.66 ± 17.79
BLOCK BOOST HC+HO (*N* = 7)	CVR (Δ%BOLD/mmHg)	0.47 ± 0.28	0.21 ± 0.16
	*r*^2^	0.46 ± 0.13	0.35 ± 0.11
	Lag (seconds)	11.66 ± 9.54	21.40 ± 11.17

*Values are in mean ± standard deviation.*

**FIGURE 4:**
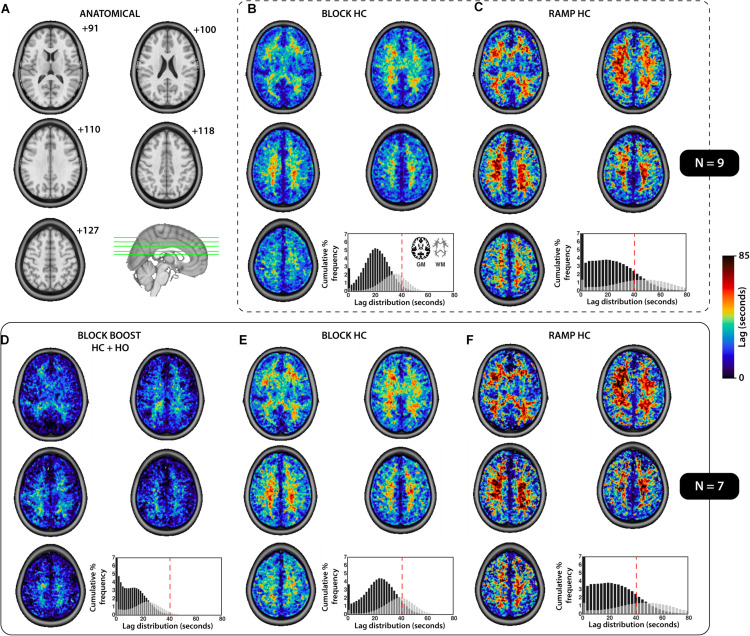
Summary of the tissue-based distribution of lag parameters for each respiratory design. **(A)** The anatomical axial slices for the MNI template providing a reference for the images presented. **(B–F)** The group-averaged lag maps (seconds) are displayed for each respiratory challenges, sub-divided based on the sample used to compute the mean image (SAMPLE.1, *N* = 9, top, **B–C**; SAMPLE.2, *N* = 7, bottom, **D–F**). The cumulative percent frequency (normalized to 100%) for the distribution of lag (seconds) is shown for each tissue which was extracted using the gray- (black) and white- (gray) matter mask displayed in **(B)**, bottom right corner. A dotted red line was added to each histogram **(B–F)** at 40 s, for reference and comparison.

While GM delays were similar between the BLOCK- and RAMP-HC (within ±2 s), longer WM lag times were observed during the RAMP-HC, when compared to the BLOCK-HC design (Table 2 and Figure 4). This was repeatable across both samples studied (Figure 4). In comparison to both HC designs, shorter GM and WM delays were observed during the BLOCK-BOOST HC+HO protocol, although the relative ∼10 s offset in the GM versus WM lag times was maintained.

The *r*^2^ fitting coefficient was similar for the BLOCK-HC and -BOOST, within both tissues (Table 2). Greater fitting estimates were observed in the RAMP-HC, compare the block designs, in both the GM and WM masks (Table 2).

## Discussion

In this study, we conducted a comprehensive analysis of the response to respiratory gas challenges in GM and WM tissues at 7T, as a way to advance our understanding of the effects that drive temporal delays in the BOLD-CVR response of healthy tissues. Findings from this study were three-fold: (1) In comparison to WM delays during the BLOCK-HC, longer WM delays were observed during the RAMP-HC suggesting that the progressive nature of the ramp stimulus may predispose the WM vasculature to a slower CVR response. We propose that this is a result of the delayed increase in the local intravascular CO_2_ gradient between blood and tissue compartments. This delayed gradient reduced the driving force facilitating the diffusion of CO_2_ into the tissues, which in turn, delayed the vasodilatory response. (2) The addition of the HO step within the HC boxcar design for the BLOCK-BOOST HC+HO protocol induced a global decrease in GM and WM delays despite maintaining a ∼10 s offset between the tissues, when compared to BLOCK-HC. We postulate that the vasoactive effect of CO_2_ within tissues remained constant and that the shortening of the delays during BOOST may have been driven by blood arrival effects (i.e., non-vasoactive) arising from the endogenous O_2_ contrast agent. (3) Comparable magnitude for lag-corrected CVR measurements were reported between the STEP-HC and RAMP-HC protocol, emphasizing that the step design is an appropriate tool to assess CVR markers, despite previous literature suggesting that a ramp-like stimulus may better model the sigmoidal relationship between changes in P_ET_CO_2_ and CBF, driving BOLD changes in signal (Bhogal et al., 2014a). Altogether, these findings support the hypothesis that differences in temporal components of CVR between vascular networks reflect the culminative effect of CO_2_ sensitivity (and/or CO_2_ diffusion rate) in local vessels, in addition to blood flow redistribution and steal effects, as previously described. Moreover, these results suggest that the addition of a BLOCK-BOOST HC+HO paradigm within clinical settings can provide insights into whether diseases causing changes in CVR do so by way of severe blood flow redistribution (which would increase blood arrival time and inflate BLOCK-BOOST HC+HO delays), or alterations in vascular properties within the vessels that could impair CO_2_ diffusion across WM tissues (i.e., atherosclerosis, amyloid angiopathy, aging-related fibrosis or stiffening of vessels; this would significantly increase the marginal gap in relative delay estimates between BLOCK-HC and BLOCK-BOOST HC+HO).

The GM and WM CVR values reported in this study are in line with previous studies looking at HC designs (Bhogal et al., 2014b, 2015) at higher spatial resolution. That said, the WM CVR values are higher than ones reported using 3T imaging (Thomas et al., 2014; Sam et al., 2016), likely due to a combination of factors including the higher BOLD CNR from 7T imaging, as well as the incorporation of temporal delay correction within the CVR computation, which has been shown to improve CVR estimates in healthy controls (Duffin et al., 2015; Poublanc et al., 2015; Donahue et al., 2016; van Niftrik et al., 2017; Champagne et al., 2019). In this study, lag-corrected WM CVR was found to be lower in the WM, across all breathing paradigms. This is consistent with existing literature (Brian, 1998; Rostrup et al., 2000; Mandell et al., 2008b; Thomas et al., 2014) suggesting that despite temporal corrections for delays in the vascular response, WM tissue may have a lower vasodilatory capacity, at least in part, due to the longer time for extravascular CO_2_ levels to build within the local vasculature, in response to global increases in arterial CO_2_ content. As originally proposed in Thomas et al. (2014) (see Figure 3), the delayed intravascular build-up in CO_2_ may be a function of the lower CBF within the WM (Duvernoy et al., 1981), such that, changes in arterial content of CO_2_ brought to the WM tissues per unit of time are comparatively lower than those for GM. This in turn increases the timespan required within which the intravascular-tissue CO_2_ gradient can drive trans-membrane diffusion of CO_2_ toward tissues, and modulate the local increases in extravascular CO_2_ (which mediates the majority of the HC-induced vasodilation).

This theory is further supported by our observation of longer delays in the WM during the RAMP-HC respiratory paradigm, in comparison to the BLOCK-HC. During a step HC paradigm, the large and rapid increase in arterial CO_2_ sets up a strong CO_2_ gradient which remains high throughout the duration of the boxcar stimulus. This effect rapidly saturates GM vessels (due to early arrival time of GM blood) and continues to flow through penetrating arteries toward downstream WM vasculature. This large increase in intravascular CO_2_ drives the concentration gradient along which CO_2_ will diffuse to induce the vasodilatory response. In other words, a high gradient leads to a faster and stronger dilatory response whereas a low gradient may induce temporal delays irrespective of blood arrival time; particularly in regions having low overall CBV such as the periventricular WM. The rapid and dynamic nature of the step also provides a setting for physiological steal mechanisms and redistribution of blood flow effects (Sobczyk et al., 2014; Bhogal et al., 2014a; Poublanc et al., 2015; Champagne et al., 2019), by which hyper-sensitive areas may respond quicker to rising levels of arterial CO_2_, and inflate temporal delays in regionally closely bound vascular territories. This may be especially significant for pathological tissues that show impaired or temporally altered hemodynamic properties due to vascular collateralization (Donahue et al., 2016).

In comparison to the BLOCK-HC, however, the RAMP-HC is assumed to be a progressive stimulus during which the vascular response has sufficient time to equilibrate with changing levels of arteriol CO_2_ content (and the corresponding blood-tissue CO_2_ gradient). Previously this was thought to lead to a more balanced response across brain regions through minimization of the dynamic redistribution (or steal) effects. This mechanisms may explain why GM delays during the RAMP were slightly higher than during the boxcar design (even though this difference was small; within ±2 s), as the slowly rising ramp stimulus allows for the vasculature to respond progressively in healthy tissues, in contrast to the dynamic STEP design which induces a rapid response in the GM vessels. In other words, healthy GM tissues may cope appropriately to rapid changes in arterial CO_2_ associated with the BLOCK-HC, resulting in relatively shorter response time (and thus, CVR delays). This warrants further exploration in clinical populations where blood flow redistribution effects are much more likely (i.e., Moyamoya disease (Conklin et al., 2010)).

Contrary to our original hypothesis, our results showed that WM CVR delays were longer during the RAMP-HC, in comparison to the BLOCK-HC. We postulate that this occurred as a result of slowly increasing intravascular CO_2_ associated with the ramp stimulus, paired with limited blood flow to the WM. Together, these effects organically delayed the build-up of higher CO_2_ gradients within WM tissues, leading to an increase in the time required to evoke the expected vascular response in the WM. This is further exaggerated by additional steal effect from GM regions that are more sensitive due to higher vascular density (and therefore more CO_2_ diffusion even considering lower CO_2_ gradients). The response in WM increases notably toward the end of the stimulus paradigm, once the ramp reaches higher P_ET_CO_2_ targets (Figure 1A) that setup stronger CO_2_ driving gradients. These findings support the original hypothesis put forward in Thomas et al. (2014) providing an explanation for the higher CVR delays in WM within healthy brain tissues, which again, may be exacerbated in settings where regional blood flow and/or CO_2_ sensitivity is disturbed.

Building on the findings from the comparison of the BLOCK- and RAMP-HC, the addition of the HO step during the HC challenge, using the BLOCK-BOOST HC+HO protocol, resulted in an overall decrease in the magnitude of delays for both the GM and WM. Despite those changes, however, the relative relationship between GM and WM was maintained during the BOOST protocol, showing a ∼10 s offset in delays between the two cerebral tissues. This supports the findings described above in that despite correction for arrival time of the stimulus into local tissues (signaled via the non-vasodilatory contrast O_2_ agent), vasodilatory response delays in the WM were consistently higher than in the GM, suggesting that this phenomenon may result from slower CO_2_ gradient build-up needed to drive diffusion into tissues. In other words, by using the BOOST protocol to compute CVR delays in clinical settings, in comparison to the BLOCK-HC delays, a more in-depth interpretation temporal response differences is possible, which teases effects related to the arrival time of the stimulus (where the magnitude of delays for BLOCK-HC and BOOST would be similar) versus local impairments in CO_2_ sensitivity within WM tissues (which would inflate the ∼10 s offset gap between the GM and WM characterized between the two methods). The vasoactive mechanisms of CO_2_ versus O_2_ may differ and it is conceivable that their interplay could influence lag estimates. The elevated CVR resulting from the BOOST HC+HO stimulus when compared to the BLOCK-HC suggests that either the vasodilatory effects of the CO_2_ overwhelmed any constrictive influence of O_2_, or the effect of increased S_v_O_2_ increased the BOLD signal beyond any HO mediated flow reductions. However, considering the relatively small O_2_ change applied in our experiments, we don’t expect appreciable constriction. Finally, the HO-induced BOLD signal is CBV-weighted, rather than reflecting CVR. This means that taken alone, the BOOST HC+HO paradigm may overestimate CVR since the HO-induced signal contribution is not explicitly accounted for. Therefore, although the BOOST HC+HO paradigm may provide additional temporal information about possible pathophysiological mechanisms, a standard CO_2_ challenge is still required for accurate CVR magnitude measurements. Disentangling respective components of the BOOST HC+HO response remains interesting for future work.

Beyond the quantitative comparison of delays across the GM and WM, results from this study show that the distribution of CVR values across respiratory paradigm were repeatable, even after adding subjects from SAMPLE.1 to SAMPLE.2. This emphasizes that voxel-based CVR measurements are consistent within the brain of healthy controls, allowing for the clinical use of such biomarker as a tool to provide reference-based voxelwise analysis of vascular impairments in patients (Sobczyk et al., 2015). Similarly, the consistency of the CVR delay distribution across the samples, between respiratory designs and within the GM and WM, supports that temporal analyses of the CVR response may too be used as a clinical tool to understand pathophysiological mechanisms associated with vascular diseases of the brain (Donahue et al., 2016; Juttukonda and Donahue, 2019).

Despite the novelty of the findings presented in this study, some limitations must be acknowledged and recognized as opportunities for future studies. First, the study employed a retroactive method to analyze datasets that were scanned as part of branching research studies. This limited our ability to synchronize and put together complete datasets for all subjects used in the analysis. Moving forward, a greater cross-sectional research design may be employed to further assess the use of the BLOCK-BOOST HC+HO protocol, and its variability across a larger sample size. Furthermore, only healthy subjects participated, limiting the external validity of these findings and preventing our ability to predict whether possible changes in arrival time, or CO_2_ sensitivity, as a result of vascular impairments, may be significant enough to be detected using the proposed methods. Future work should therefore consider implementing these analyses in clinical population with known vascular diseases, in order to assess the potential utility of such combined approach. Specifically, clinical studies may consider studying the effect of pathological disease mechanisms on the local diffusion rate of CO_2_ into tissues, which could be tested using a combination of the BLOCK-HC and BLOCK BOOST HC+HO. It may be hypothesized that local damages to the endothelium may manifest as increases in the discrepancy between the GM and WM CVR lag times observed under each respiratory challenge, while blood arrival remains relatively unchanged (and thus delays would still be shorter under BLOCK BOOST HC+HO). Finally, the magnitude of the delivered BLOCK-HC change in P_ET_CO_2_ was smaller than the maximum peak change in P_ET_CO_2_ reached during the RAMP-HC, which may impact the values extracted for delays across tissues, given that the greater stress on local vasculature could promote a micro-environment within which blood flow redistribution effects are more apparent across regions with differences in hemodynamic capacity. Thus, future work may also consider examining the effect of changing the magnitude of the step stimulus and exploring whether larger CO_2_ gradient changes the voxelwise lag maps.

## Conclusion

As highlighted in this study, tissue-based differences in CVR and temporal markers for the response to hypercapnia are governed by a number of factors that reflects a compounded effect dependent on arterial arrival time, CO_2_ sensitivity and CO_2_ diffusion rate, blood flow redistribution, and steal effects, and vascular response speed. Here, we provide evidence showing that differences in the temporal components of CVR may be influenced by intravascular CO_2_ gradients in local vessels, which, in addition to membrane permeability, and blood flow effects, determines the rate of diffusion within local tissues that then drives the vasodilatory response. The proposed mechanisms presented in this study, in support of Thomas et al. (2014), may provide a partial explanation for the slower WM response to hypercapnia in comparison to GM, in healthy tissues. Finally, the results presented suggest that the implementation of a BLOCK BOOST HC+HO respiratory challenge may provide additional insight about possible pathophysiological mechanisms underlying vascular diseases based on whether they are driven by severe blood flow redistribution, or alterations in vascular properties within the vessels that would affect trans-membrane CO_2_ diffusion into tissues. Moving forward, the combination of a BLOCK-HC and BLOCK-BOOST respiratory paradigms may be used to untangle these factors driving CVR and the speed of response, as a tool to help to improve the diagnosis, prognosis and management of patients with vascular brain diseases.

## Data Availability Statement

The raw data supporting the conclusions of this article will be made available by the authors, without undue reservation.

## Ethics Statement

The studies involving human participants were reviewed and approved by WMO (Wet Medisch Wetenschappelijk Onderzoek). The patients/participants provided their written informed consent to participate in this study.

## Author Contributions

AB was responsible for all the data collection and data pre-processing. AC ran the data analysis and wrote the manuscript. AB and AC edited and reviewed the manuscript and prior to submission. Both authors contributed to the article and approved the submitted version.

## Conflict of Interest

The authors declare that the research was conducted in the absence of any commercial or financial relationships that could be construed as a potential conflict of interest.
