# Supplementary material for: Insights Into Cerebral Tissue-Specific Response to Respiratory Challenges at 7T: Evidence for Combined Blood Flow and CO2-Mediated Effects
Source: Front Physiol. 2021 Jan 28;12:601369. doi: 10.3389/fphys.2021.601369 (PMC7876301; doi:10.3389/fphys.2021.601369)
Supplement: Supplementary file 1 [file Table_1.DOCX]

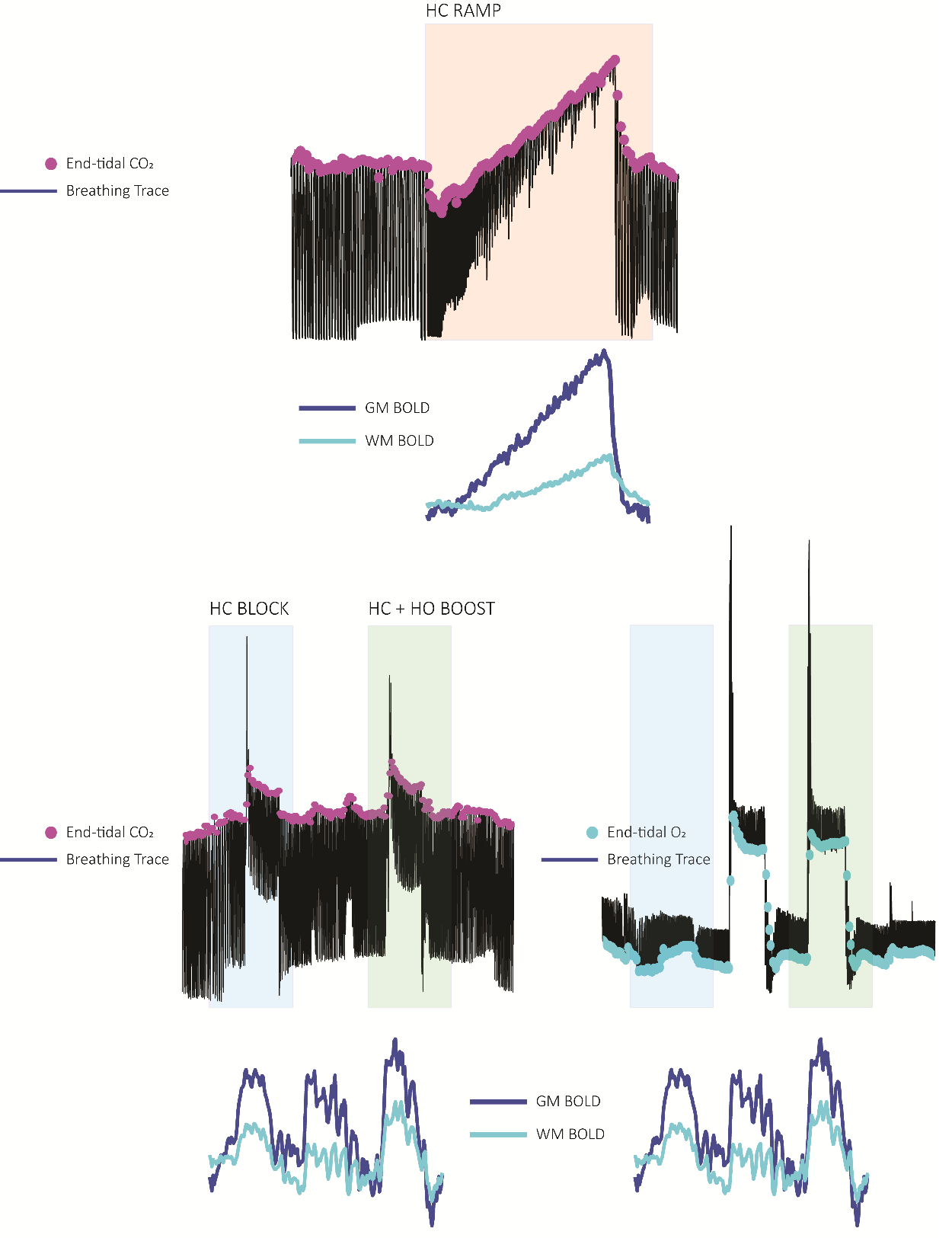


**Supplemental Figure 1.** **TOP**: Progressive RAMP-HC stimulus performed under normoxic conditions (orange shaded). The corresponding GM and WM BOLD signal responses are depicted below the end-tidal trace. Depicted BOLD signals have been normalized to percent change. No further post-processing has been applied (i.e., denoising, smoothing etc.); **BOTTOM**: BLOCK-HC (blue shaded) and HC + HO BOOST (green shaded) stimuli for a single subject run are depicted. Note the normoxic stimuli during the BLOCK-HC. Corresponding GM and WM ROI BOLD responses are shown below end-tidal traces. CVR is calculated as the voxel-wise regression of the %BOLD signal change against the corresponding end-tidal CO_2_ trace (see Figure 3 in the manuscript). BOLD = blood oxygen level dependent, GM = grey-matter, HC = hypercapnia, WM = white-matter
